# Cook County Medical Society

**Published:** 1853-04

**Authors:** DeLaskie Miller

**Affiliations:** Secretary


*Cook County Medical Society.*

At the Annual meeting of the Cook County Medical Society, held in the City of Chicago, on Tuesday, April 5th, 1853, the following named persons were elected officers for the ensuing year, viz:—Dr. Wm. B. Herrick, President; Dr. N. S. Davis, Vice President, and Dr. DeLaskie Miller, Secretary. The following persons were elected Delegates to the American Medical Association, viz:—Drs. Wm. B. Herrick, Horatio Hitchcock and Erial McArthur.

On motion, the Delegates to the American Medical Association were aothorized to appoint substitutes from the members of this Society.

The Delegates elected to the State Medical Society, were Drs. J. V. Z. Blaney, DeLaskie Miller, J. H. Bird, Erial McArthur, and Wm. E. Clark.

On motion of Dr. Blaney, the Delegates to the State Medical Society were authorized to appoint substitutes.

The Society then adjourned.

DeLaskie Miller, Secretary.

